# Supplementary material for: Adaptation to cystine limitation stress confers a targetable lipid metabolism vulnerability in pancreatic ductal adenocarcinoma
Source: Nat Commun. 2025 Dec 31;17:1343. doi: 10.1038/s41467-025-68099-0 (PMC12872613; doi:10.1038/s41467-025-68099-0)
Supplement: Supplementary file 1 — Supplementary Information [file 41467_2025_68099_MOESM1_ESM.pdf]

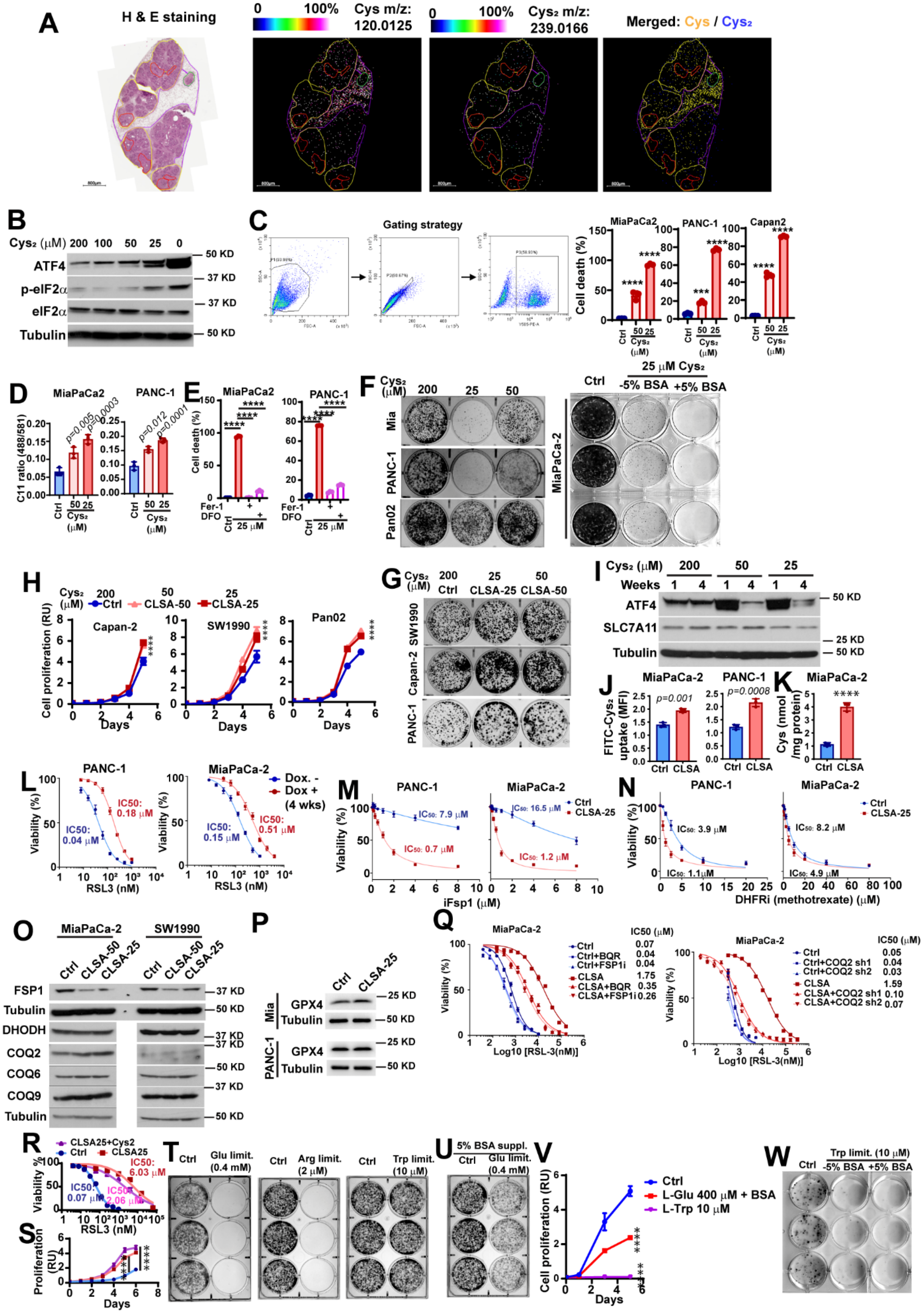

A
H & E staining
Cys m/z: 120.0125
Cys₂ m/z: 239.0166
Merged: Cys / Cys₂
B
Cys₂ (μM) 200 100 50 25 0
ATF4
p-eIF2α
eIF2α
Tubulin
50 KD
37 KD
37 KD
50 KD
C
Gating strategy
MiaPaCa2
PANC-1
Capan2
Cell death (%)
Ctrl
50 25
Cys₂ (μM)
D
MiaPaCa2
PANC-1
C11 ratio (488/581)
p=0.005
p=0.0003
p=0.012
p=0.0001
E
Cell death (%)
Fer-1
DFO
25 μM
F
Cys₂ (μM)
200 25 50
Mia
PANC-1
Pan02
Ctrl
25 μM Cys₂
-5% BSA
+5% BSA
MiaPaCa-2
G
Cys₂ (μM)
200 Ctrl
25 CLSA-25
50 CLSA-50
SW1990
Capan-2
PANC-1
H
Cys₂ (μM)
200 Ctrl
50 CLSA-50
25 CLSA-25
Capan-2
SW1990
Pan02
Cell proliferation (RU)
Days
I
Cys₂ (μM) 200 50 25
Weeks 1 4 1 4 1 4
ATF4
SLC7A11
Tubulin
50 KD
25 KD
50 KD
J
MiaPaCa-2
PANC-1
p=0.001
p=0.0008
FITC-Cys₂ uptake (MFI)
Ctrl CLSA
K
MiaPaCa-2
Cys (nmol /mg protein)
Ctrl CLSA
L
PANC-1
MiaPaCa-2
Dox. -
Dox + (4 wks)
IC50: 0.18 μM
IC50: 0.04 μM
IC50: 0.51 μM
IC50: 0.15 μM
Viability (%)
RSL3 (nM)
M
PANC-1
MiaPaCa-2
Ctrl
CLSA-25
IC50: 7.9 μM
IC50: 0.7 μM
IC50: 16.5 μM
IC50: 1.2 μM
Viability (%)
iFsp1 (μM)
N
PANC-1
MiaPaCa-2
Ctrl
CLSA-25
IC50: 3.9 μM
IC50: 1.1 μM
IC50: 8.2 μM
IC50: 4.9 μM
Viability (%)
DHFRi (methotrexate) (μM)
O
MiaPaCa-2
SW1990
Ctrl CLSA-50 CLSA-25
FSP1
Tubulin
DHODH
COQ2
COQ6
COQ9
Tubulin
37 KD
50 KD
37 KD
37 KD
50 KD
37 KD
50 KD
P
Ctrl CLSA-25
Mia
GPX4
Tubulin
PANC-1
GPX4
Tubulin
25 KD
50 KD
25 KD
50 KD
Q
MiaPaCa-2
IC50 (μM)
Ctrl 0.07
Ctrl+BQR 0.04
Ctrl+FSP1i 0.04
CLSA 1.75
CLSA+BQR 0.35
CLSA+FSP1i 0.26
Viability (%)
Log10 [RSL-3(nM)]
MiaPaCa-2
IC50 (μM)
Ctrl 0.05
Ctrl+COQ2 sh1 0.04
Ctrl+COQ2 sh2 0.03
CLSA 1.59
CLSA+COQ2 sh1 0.10
CLSA+COQ2 sh2 0.07
Viability (%)
Log10 [RSL-3(nM)]
R
CLSA25+Cys2
Ctrl
CLSA25
IC50: 6.03 μM
IC50: 0.07 μM
IC50: 2.06 μM
Viability %
RSL3 (nM)
S
Proliferation (RU)
Days
T
Ctrl
Glu limit. (0.4 mM)
Ctrl
Arg limit. (2 μM)
Ctrl
Trp limit. (10 μM)
U
5% BSA suppl.
Ctrl
Glu limit. (0.4 mM)
V
Ctrl
L-Glu 400 μM + BSA
L-Trp 10 μM
Cell proliferation (RU)
Days
W
Ctrl
Trp limit. (10 μM)
-5% BSA
+5% BSA

Supplementary Figure 1. Adaptation to Cys_2 limitation stress promotes PDAC cell proliferation.

A, Representative MALDI-MS images and corresponding H&E staining showing the distribution of Cys and Cys_2 in different regions pancreatic tissues from 8-weeks old KFC mice. Red, yellow, green and purple indicated regions of PDAC, PanIN, normal pancreatic acinar and tumor infiltrating lymphocytes, respectively.

B, The effects of Cys_2 limitation (24 hours) on the levels of ATF4, p-eIF2α in PANC-1 cells.

C, The effects of 72 hour CLS (50 μM or 25 μM) on PDAC cell death, as determined by PI staining and flow cytometry. Left, gating strategy for PI staining and flow cytometry; right, quantitation of flow cytometry data.

D, The effects of 72 hour CLS (50 μM or 25 μM) on lipid peroxidation in PDAC cells, as determined by C11-BODIPY staining.

E, The effects of DFO (100 μM) and ferrostatin-1 (1 μM) treatment on cell death in PDAC cells after 72-hour CLS (25 μM) treatment.

F, Left, the effects CLS (25 or 50 μM) on colony formation in naïve PDAC cells; right, the effect of 5% BSA supplementation on naive MiaPaCa-2 colony formation in CLS (25 μM Cys_2) media.

G, Colony formation by CLSA-25 (in 25 μM Cys_2), CLSA-50 (in 50 μM Cys_2) PDAC cells and their corresponding naïve control PDAC cells (in 200 μM Cys_2).

H, The effects of CLSA on cell proliferation in Capan-2, SW1990 and Pan02 cells.

I, The Western blotting analysis of the levels of ATF4 and SLC7A11 in MiaPaCa-2 cells after culture in cystine replete (200 μM) or cystine limited (25 or 50 μM) media for 1 or 4 weeks.

J, FITC-cystine uptake by control of CLSA25 MiaPaCa-2 and PANC-1 cells, as determined by flow cytometry.

K, Control or CLSA-25 MiaPaCa-2 cells were cultured in cystine replete (200 μM) media for 24 hours and the levels of intracellular cysteine were determined through LC-MS/MS after NEM derivatization.

L, The effects of chronic SLC7A11 knockdown (dox+, 4 weeks) on sensitivities to RSL-3 induced ferroptosis in PANC-1 and MiaPaCa-2 cells.

M and N, The sensitivities of control or CLSA-25 PANC-1 and MiaPaCa-2 cells to iFSP1 (M) or methotrexate (N) induced cell death.

O and P, The expression levels of FSP1, DHODH, COQ2, COQ6, COQ9 (O) and GPX4 (P) in control or CLSA SW1990 and MiaPaCa-2 cells.

Q, The effects of BQR (20 μM), iFSP1 (0.1 μM) or COQ2 knockdown on sensitivities to RSL-3 induced ferroptosis in control or CLSA-25 MiaPaCa-2 cells.

R and S, The RSL-3 sensitivities (R) and cell proliferation (S) in MiaPaCa-2 control, CLSA-25 and CLSA-25 cells cultured in cystine replete (200 μM) media (CLSA25+Cys_2) for 4 weeks were determined.

T, The effects of glutamine limitation (400 uM), arginine limitation (2 uM) and tryptophan limitation (10 uM) on colony formation (2 weeks) in MiaPaCa-2 cells.

U, The effects of glutamine limitation (400 uM) and BSA supplementation (5%) on MiaPaCa-2 colony formation (2 weeks).

V, MiaPaCa-2 cells were cultured in conventional DMEM media or Glu-limited DMEM (0.4 mM Glu plus 5% BSA) or Trp-limited DMEM (10 uM Trp) for 4 weeks before being used for cell proliferation assay in their respective nutrient replete or nutrient-limited cell culture media.

W, Control MiaPaCa-2 cells or MiaPaCa-2 cells after 2-week exposure to Trp-limited (10 μM) media were harvested and used for colony formation assay in regular DMEM media or Trp-limited media (10 μM) with or without 5% BSA supplementation.

Data in (C-E) were analyzed using two sample, two-tailed paired t-test. Data in (H), (S) and (V) were analyzed using two-way ANOVA followed by Tukey's multiple comparison test. **** represents $p < 0.0001$. All error bars are mean ±SD

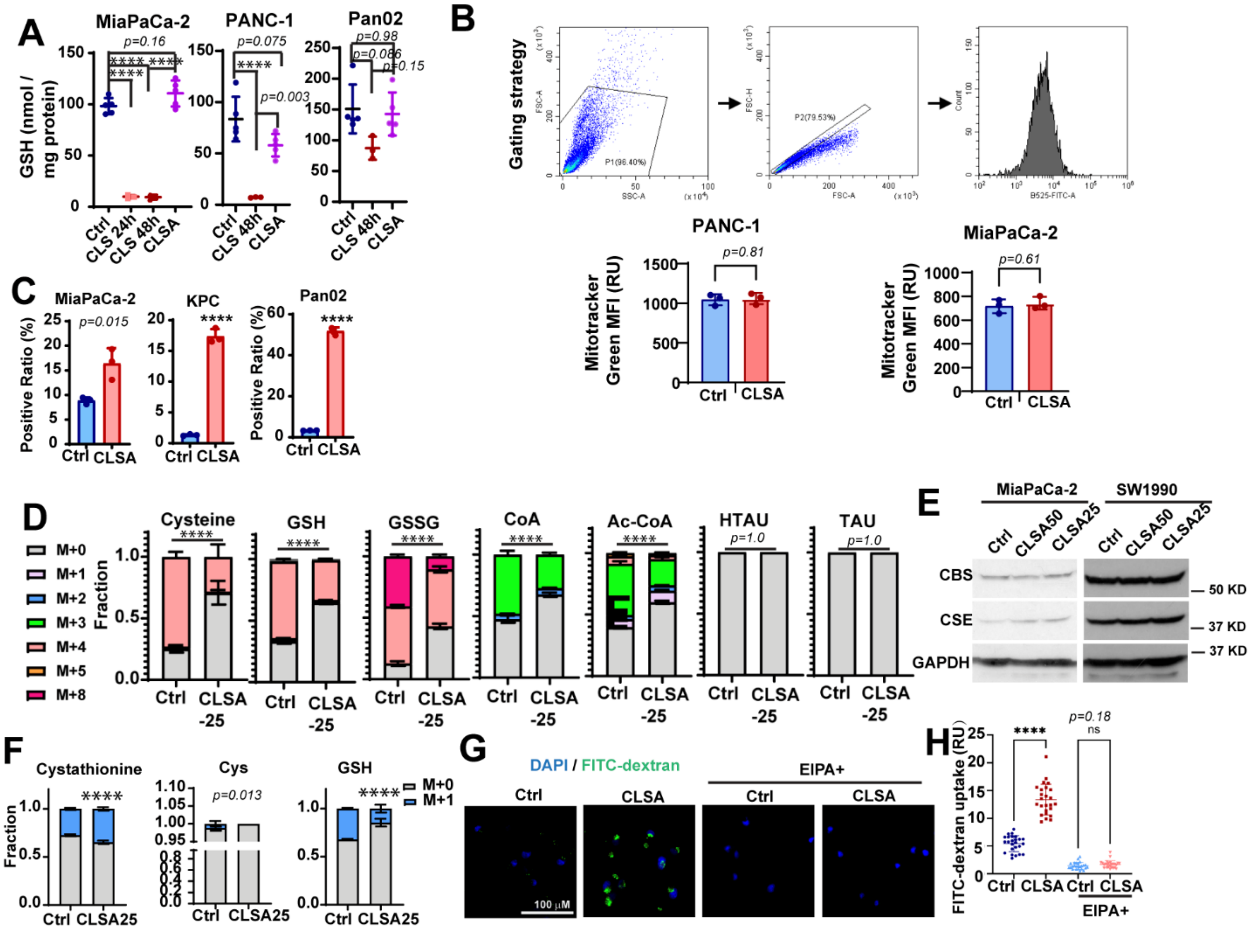

Supplementary Figure 2. Cys metabolism in CLSA PDAC cells.

A, GSH levels in PDAC cells after being cultured in Cys_2-limited (25 uM) media for 24-48 hours, or after adaptation to 25 uM Cys_2-limitation for 4 weeks were determined through LC-MS/MS.

B, Mitochondria volume in control or CLSA-25 PANC-1 and MiaPaCa-2 cells were determined through Mitrotracker Green staining and flow cytometry. Upper panel, gating strategy for Mitotracker Green flow cytometry. Lower panel, quantitation of flow cytometry data.

C, 2-NBDG assay uptake the levels of 2-NBDG positive cells in control or CLSA-25 MiaPaCa-2, KPC and Pan02 cells after 30 minute incubation with 2-NBDG.

D, Steady state labeling of Cys metabolites after incubation with [$^{13}C_6$, $^{15}N_2$]-Cys_2 for 24 hours.

E, The Western blotting analysis of the levels of CBS and CSE in control or CLSA MiaPaCa-2 and SW1990 cells.

F, Control or CLSA-25 MiaPaCa-2 cells were incubated with [1-^{13}C]-Serine for 4 hours and the fractional labeling of cystathionine, Cys and GSH were determined through LC-MS/MS.

G and H, Representative confocal microscopy images (G) and quantitation (H) showing the macropinocytosis of FITC-dextran by control or CLSA-25 MiaPaCa-2 cells with or without micropinocytosis inhibitor EIPA (25 μM).

Data in (A-D), (F) and (H) were analyzed using two sample, two-tailed, t-test. **** represents p< 0.0001. All error bars are mean ±SD

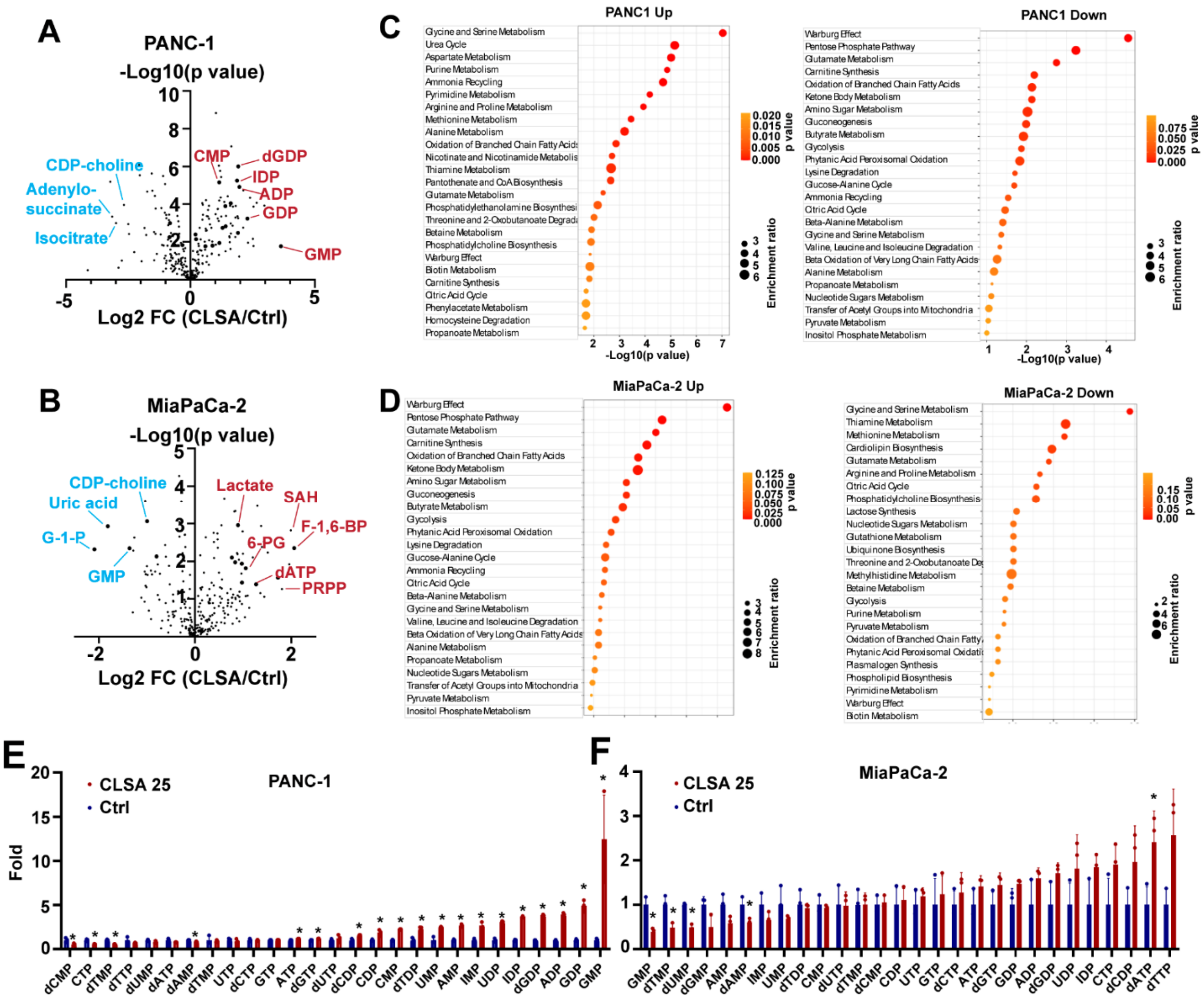

Supplementary Figure 3. Elevated nucleotide levels in CLSA PDAC cells.

A and B, Scatter dot plots showing the effect of CLSA-25 on metabolomics reprograming in PANC-1 (A) or MiaPaCa-2 (B) cells.

C and D, MSEA analysis of upregulated and downregulated metabolites in PANC-1 (C) and MiaPaCa-2 cells (D).

E and F, Bar graph showing the nucleotide levels in metabolomics screening using control or CLSA-25 PANC-1 (E) or MiaPaCa-2 (F) cells.

Data in (A), (B), (E) and (F) were analyzed using two sample, two-tailed, t-test without multiple comparison correction. * indicates $p<0.05$. All error bars are mean ±SD

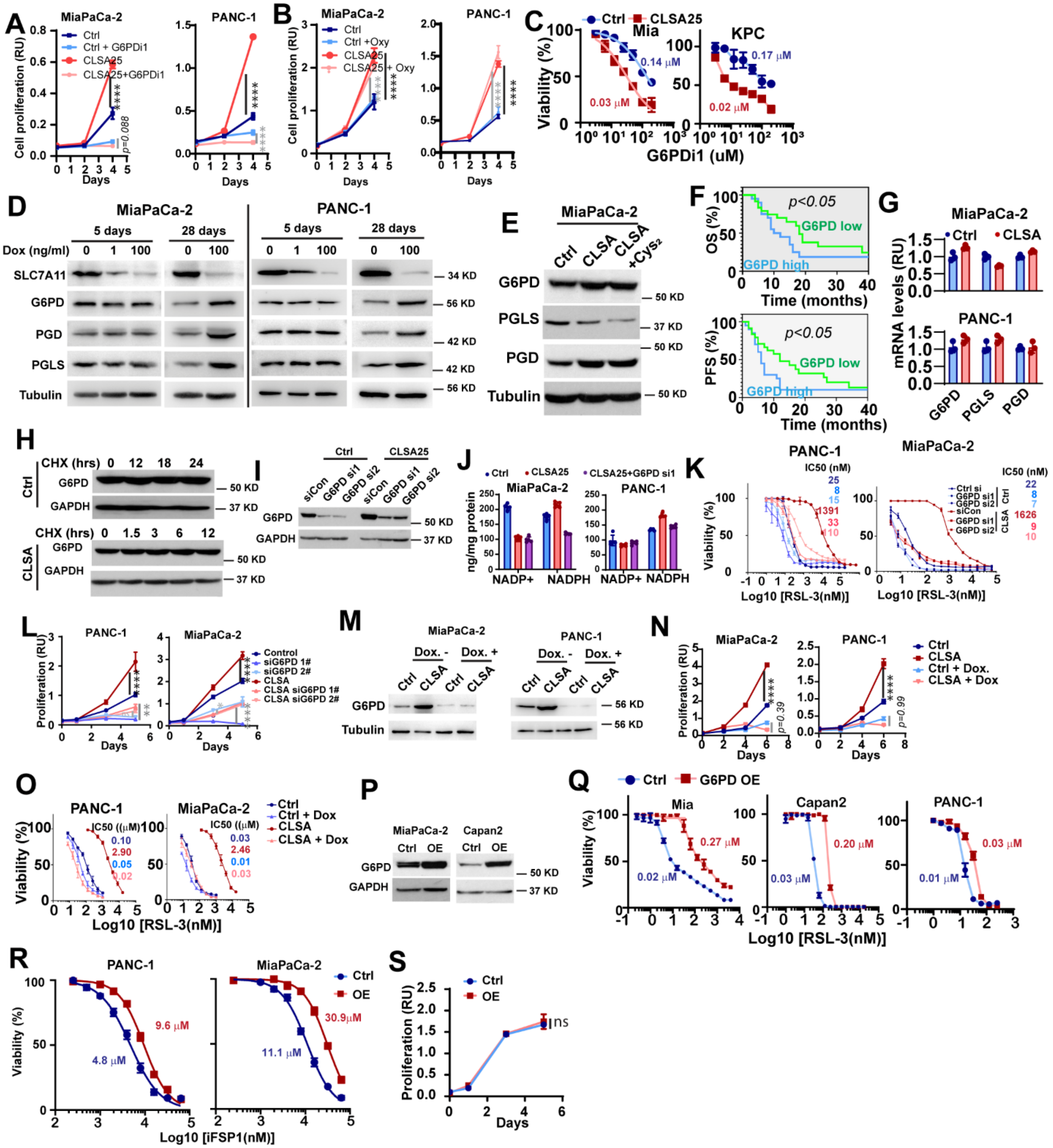

Supplementary Figure 4. OxPPP is required for CLSA-mediated cell proliferation and ferroptosis resistance.

A and B, The effects of OxPPP inhibitor G6PDi1 (50 µM) and reductive PPP inhibitor oxythiamine (50 µM) treatment on cell proliferation in control or CLSA-25 PANC-1 and MiaPaCa-2 cells.

C, The effect of G6PDi1 treatment on cell viability in control or CLSA-25 MiaPaCa-2 and KPC cells.

D, Western blotting analysis of the levels of SLC7A11, G6PD, PGLS and PGD in MiaPaCa-2 and PANC-1 cells after doxycycline-induced knockdown of SLC7A11 for 5 (left) or 28 (right) days.

E, Western blotting analysis of the levels of G6PD, PGLS and PGD in MiaPaCa-2 control, CLSA-25 and CLSA-25 cells cultured in cystine replete media (CLSA+Cys_2) for 28 days.

F, Kaplan-Meier analysis of the overall survival (OS) and progression free survival (PFS) of G6PD-high or G6PD-low PDAC patients (n=100).

G, mRNA transcript levels of G6PD, PGLS and PGD in control and CLSA-25 MiaPaCa-2 and PANC-1 cells.

H, The effect of cycloheximide (100 ug/mL) treatment on protein levels of G6PD in control or CLSA-25 MiaPaCa-2 cells.

I, The effect of G6PD siRNA1 and 2 on the protein levels of G6PD in control or CLSA-25 MiaPaCa2 cells.

J, NADPH and NADP+ in MiaPaCa-2 and PANC1 cells used for the calculation of NADPH/NADP+ ratios in Fig. 4J.

K, The effects of G6PD siRNA knockdown on sensitivities to RSL-3 induced ferroptosis in control and CLSA-25 PANC-1 and MiaPaCa-2 cells.

L, The effects of G6PD siRNA knockdown on cell proliferation in control and CLSA-25 PANC-1 and MiaPaCa-2 cells.

M, The effects of doxycycline-induced G6PD shRNA (3 Days) on the protein levels of G6PD in control or CLSA-25 MiaPaCa-2 cells.

N and O, The effects of doxycycline-induced G6PD knockdown on cell proliferation (N) and RSL-3 induced ferroptosis (O) in control or CLSA-25 PANC-1 and MiaPaCa-2 cells.

P, G6PD expression levels in PDAC cells stably expressing pLX304-vector control or pLX304-G6PD.

Q-S, The effects of G6PD overexpression (OE) on sensitivities to RLS-3 (Q), iFSP1 (R) or cell proliferation (S) in PDAC cells.

Data in (A), (B), (L), (N) and (S) were analyzed using two-way ANOVA followed by Tukey's multiple comparison test. **** represents $p < 0.0001$. All error bars are mean ±SD

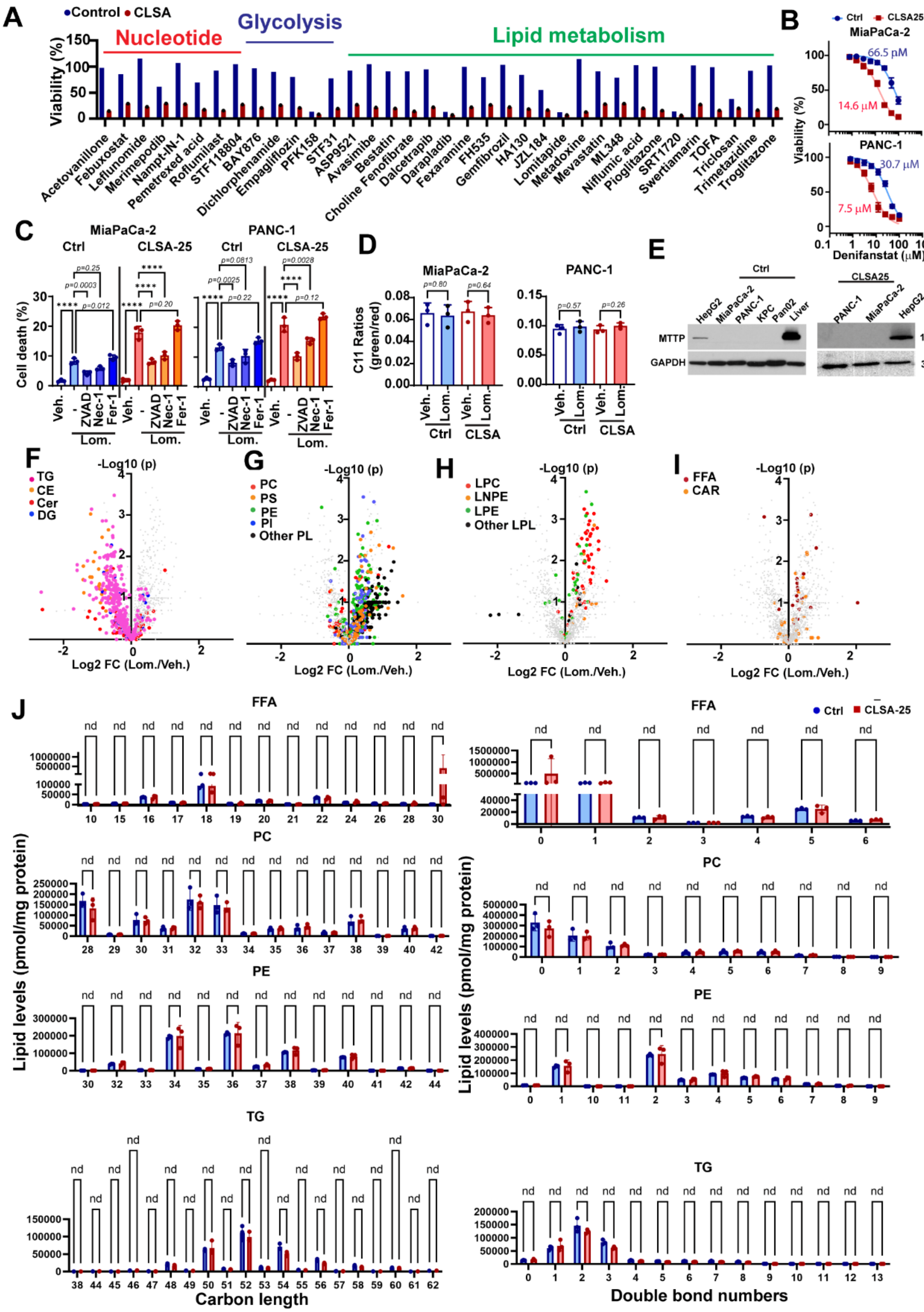

Supplementary Figure 5. Lipid metabolism vulnerability in CLSA PDAC.

A, Summary of cell viability data from positive hits in HTS drug screening.

B, Sensitivities of control or CLSA-25 MiaPaCa-2 and PANC-1 cells to FASN inhibitor (Denifanstat) and SCD1 inhibitor (A939572). The IC_{50} for A939572 was not determined.

C, The effects of apoptosis inhibitor ZVAD (50 μM), necroptosis inhibitor necrostatin-1 (10 μM) and ferroptosis inhibitor ferrostatin-1 (1 μM) on lomitapide (2 μM)-induced cell death.

D, The effects of lomitapide treatment (2 μM, 24 hrs) on lipid peroxidation levels in control and CLSA-25 PDAC cells, as determined by C11 BODIPY fluorescence ratios.

E, Western blotting showing the levels of MTTP protein in naïve control or CLSA-25 human and murine PDAC cells. HepG2 and mouse liver lysate were used as positive control.

F-I, Volcano plots showing indicated the effects of lomitapide treatment (1 μM, 24 hrs) on changes in neutral lipids (F), phospholipids (G), lysophospholipids (H) and toxic lipid species (I) in CLSA-25 MiaPaCa-2 cells.

J, Analysis of the effects of lomitapide treatment on carbon length and double bond numbers in FFA, PC, PE and TG.

Data in (C) and (D) were analyzed using one way ANOVA followed by Dunnett's multiple comparison test. **** represents $p < 0.0001$. Data in (J) were analyzed using unpaired multiple t-test and * indicated false discovery rate $q<0.05$. Data in (F-I) were analyzed using two-tailed t-test without multiple comparison correction. All error bars are mean ±SD

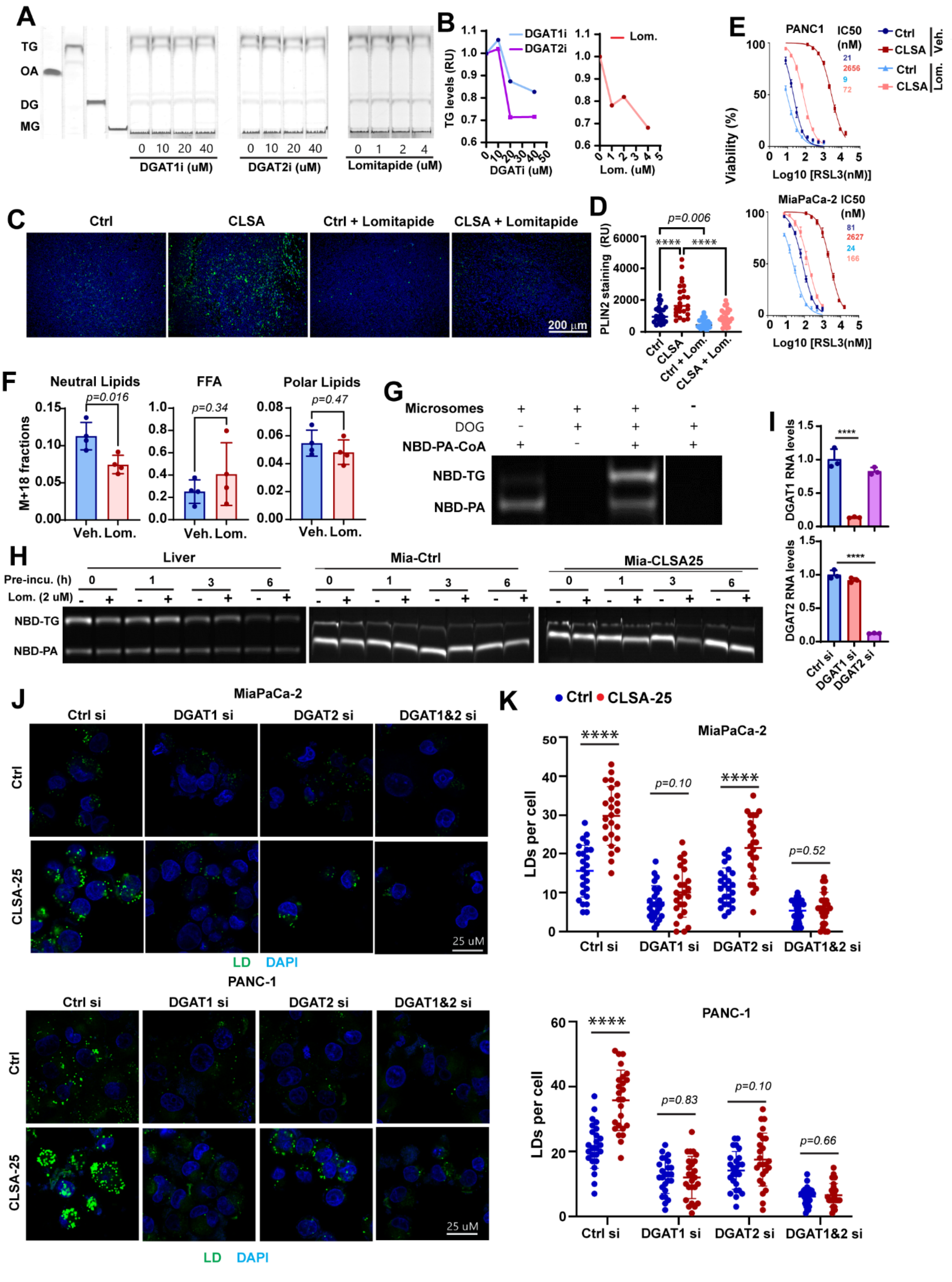

Supplementary Figure 6. Inhibition of LD and TG synthesis by lomitapide.

A and B, TLC analysis (A) and densitometry analysis (B) showing the dose-dependent effects of DGAT1i (A922500) and DGAT2i (PF-06424439) and lomitapide treatment on TG levels in MiaPaCa-2 cells.

C and D, Representative PLIN2 immunofluorescence staining images (C) and quantitation of staining intensities (D) of FFPE sections using xenograft tumor tissues harvested from control or CLSA-25 MiaPaCa-2 xenograft experiments with or without lomitapide treatment (10 mg/kg).

E, The effects of lomitapide treatment (0.5 μM) on RSL-3 induced ferroptosis in PANC-1 or MiaPaCa-2 cells.

F, The effects of lomitapide treatment (2 μM) on [U-^{13}C]-Oleate incorporation into neutral lipids, FFA and polar lipids in naive MiaPaCa-2 cells.

G, TLC analysis showing the synthesis of NBD-TG in the presence of DOG (200 uM), NBD-PA-CoA (25 μM) and liver microsome (5 μg protein).

H, The effects of pre-incubation time on the inhibitory activities of lomitapide in the *in vitro* DGAT activity assay in (G). Pre-incubation of lomitapide with microsome membrane for 1 hour or longer completely abrogated the inhibitory activities of 2 μM lomitapide.

I, DGAT1 siRNA and DGAT2 siRNA on the mRNA transcript levels of DGAT1 and 2 in MiaPaCa-2 and PANC-1 cells.

J and K, Representative confocal micrograph (J) and quantatitation (K) showing the effects of the effects of DGAT1 and DGAT2 knockdown on LD levels in control or CLSA-25 MiaPaCa-2 cells.

Data in (F), (I) and (K) were analyzed using two-tailed t-test. **** represents $p < 0.0001$. All error bars are mean ±SD

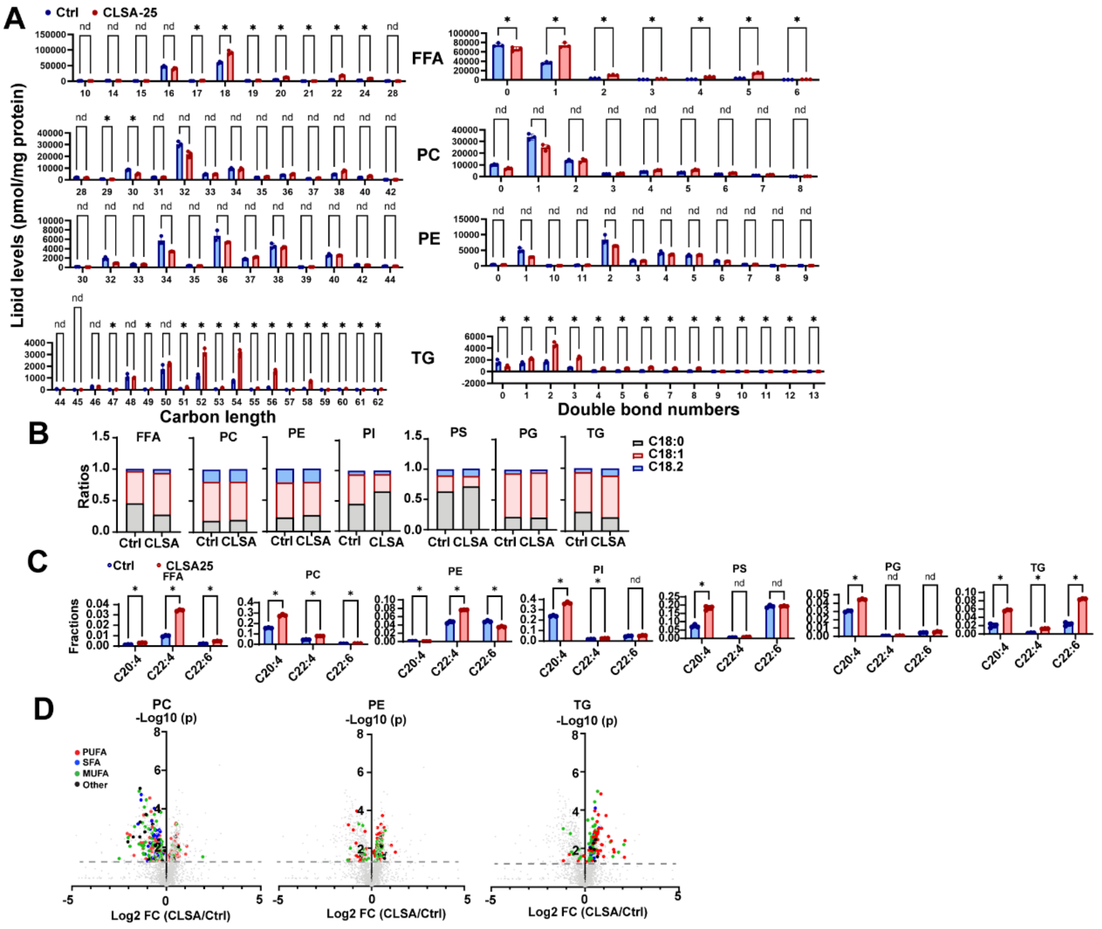

Supplementary Figure 7. The effect of CLSA on lipidomics reprograming in PANC-1 and MiaPaCa-2 cells.

A, Analysis of carbon length and double bond numbers in FFA, PC, PE and TG in control and CLSA-25 PANC-1 cells.

B, Analysis C18:0/C18:1/C18:2 ratios in several major lipid classes (FFA, PC, PE, PI, PS, PG and TG) in control and CLSA-25 PANC-1 cells.

C, The relative abundance arachidonic acid (C20:4), adrenic acid (C22:4) and DHA (C22:6) in major lipid classes (FFA, PC, PE, PI, PS, PG and TG) in control and CLSA-25 PANC-1 cells.

D, Scatter dot plots showing the PC, PE and TG species containing PUFA, SFA and MUFA.

Data in (A) and (C) were analyzed using unpaired multiple t-test. * indicated false discovery rate q<0.05. Data in (D) were analyzed using two-sided t-test without multiple comparison correction. All error bars are mean ±SD

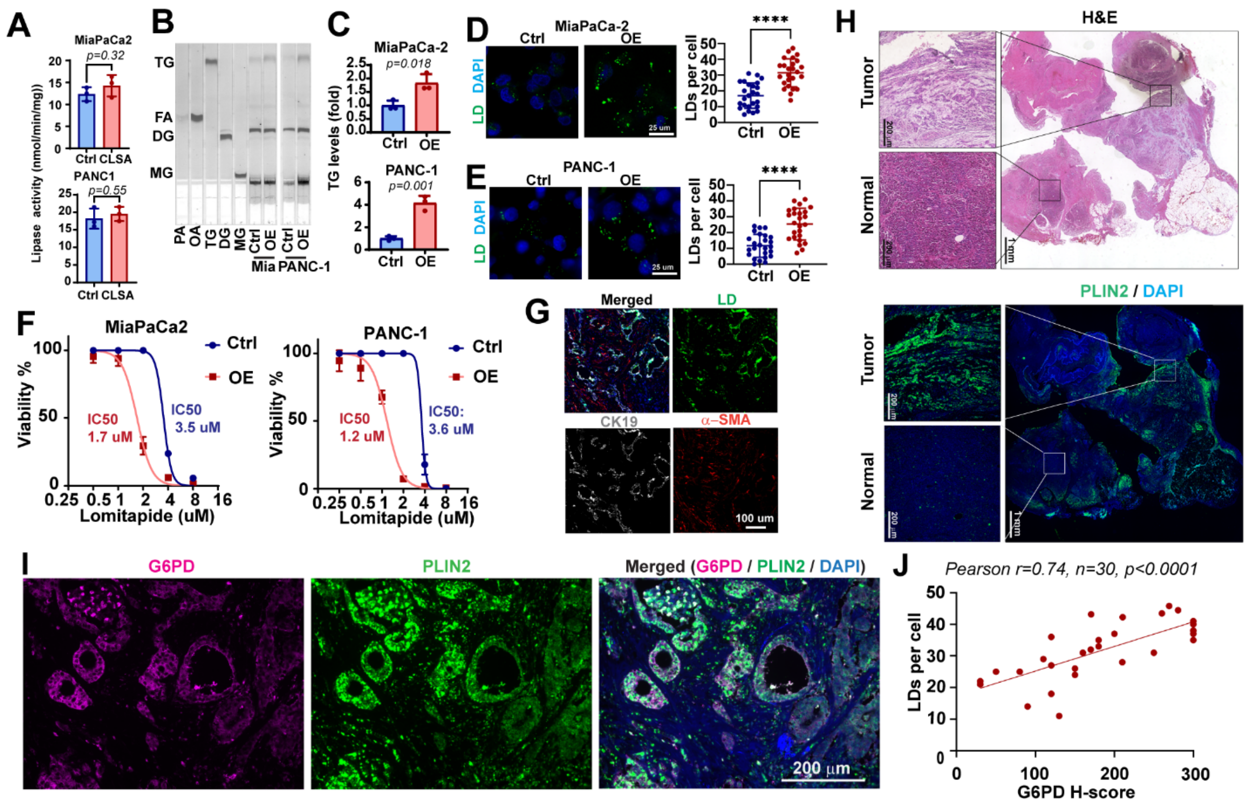

Supplementary Figure 8. The role of G6PD in LD formation.

A, Lipase activities in control or CLSA-25 MiaPaCa-2 and PANC-1 cells.

B and C, TLC analysis (B) and densitometry quantitation (C) showing the effects of G6PD overexpression on TG levels in MiaPaCa-2 and PANC-1 cells.

D and E, Representative confocal micrographs (left) and quantitation (right) showing the effects of CLSA-25 on LD levels in MiaPaCa-2 (D) and PANC-1 (E) cells.

F, The effects of G6PD overexpression on sensitivities to lomitapide treatment in MiaPaCa-2 and PANC-1 cells.

G, LD staining in tumor cells (CK19) and cancer associated fibroblasts (α-SMA) in PDAC patient tissue sections. (scale bar ??)

H, PLIN2 staining and H&E staining of FFPE sections from PDAC patients showing the LD levels in PDAC (tumor) and normal tissues from the pancreas of the same patient.

I, PLIN2 and G6PD immunofluorescence staining and representative confocal microscopy images showing the correlation between LD and G6PD expression in PDAC patient tissues.

J, Pearson's correlation analysis showing the correlation between G6PD and PLN2 LD staining in 30 PDAC patients.

Data in (A), (C) and (D) were analyzed using two-tailed, two sample, unpaired Student's t-test. Data in (J) were analyzed using Pearson's correlation analysis. **** represents p< 0.0001, respectively. All error bars are mean ±SD

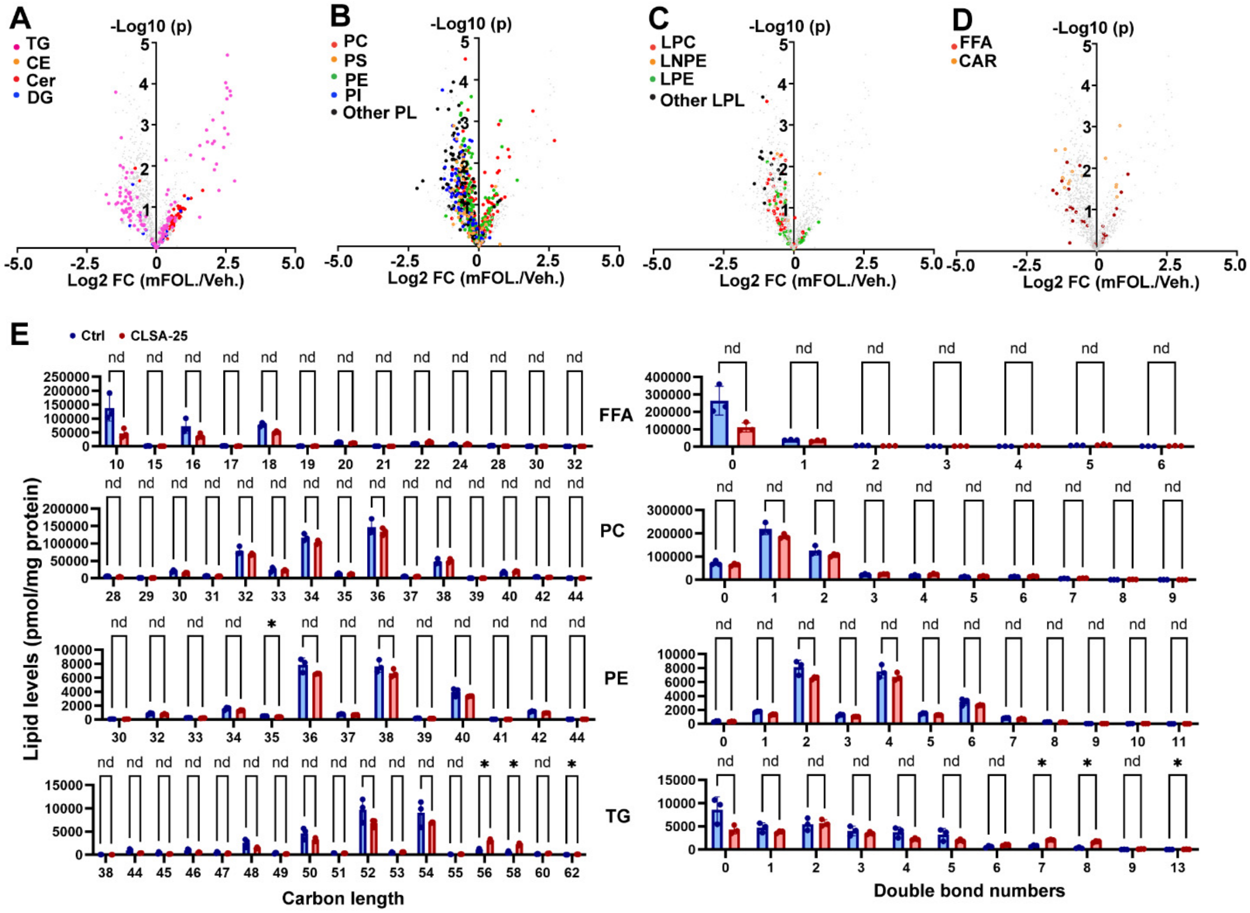

Supplementary Figure 9. The effect of mFOLFIRINOX treatment on lipidomics changes in MiaPaCa-2 cells.

A-D, Scatted dot plots showing the effects of mFOLFIRINOX treatment on neutral lipids (A), phospholipids (B), lysophospholipids (C) and toxic lipids (D) in naïve MiaPaCa-2 cells.

E, Analysis of carbon length and double bond numbers in FFA, PC, PE and TG in control and CLSA-25 PANC-1 cells.

Data in (E) were analyzed using unpaired multiple t-test. * indicated false discovery rate q<0.05. Data in (A-D) were analyzed using two-sided t-test without multiple comparison correction. All error bars are mean ±SD

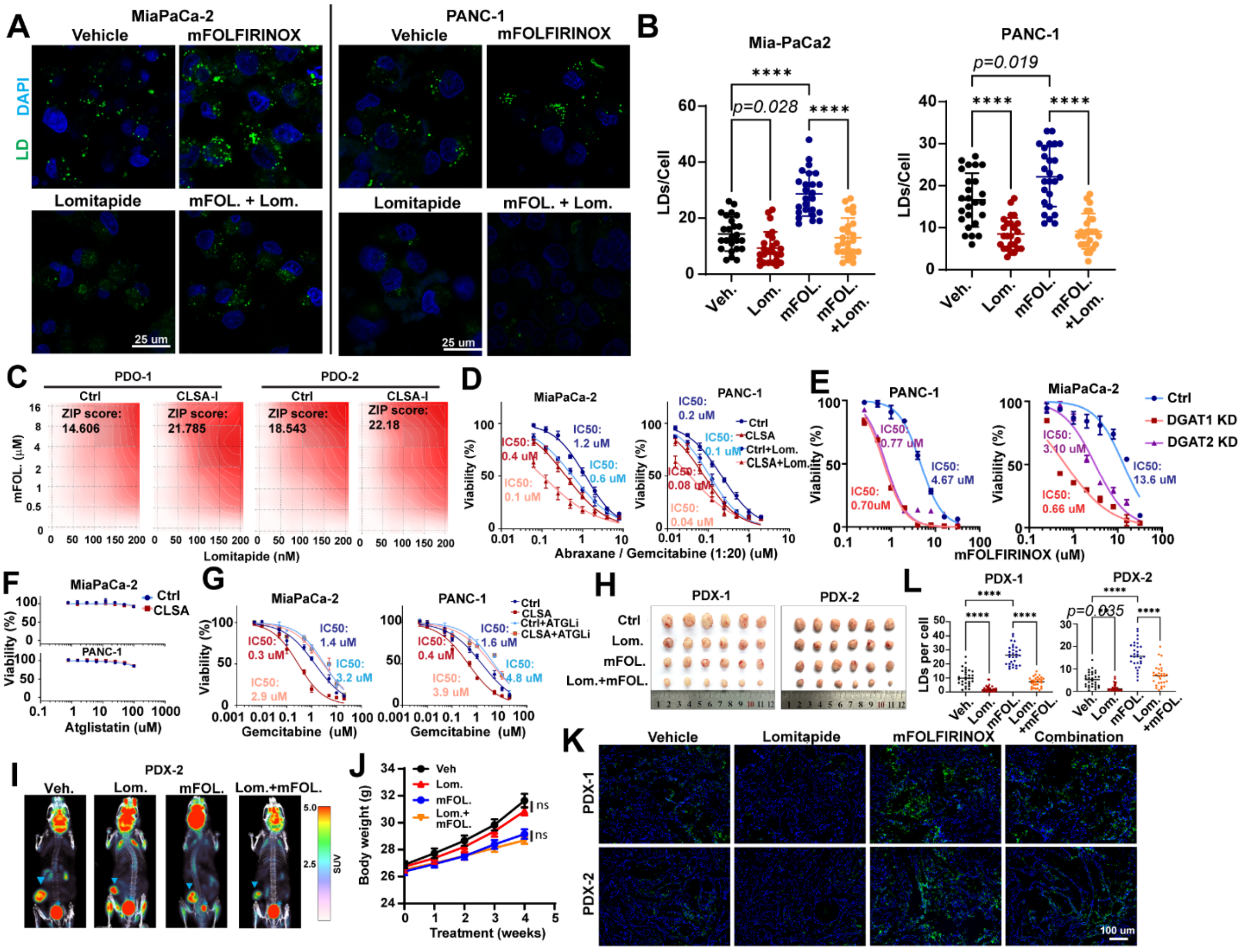

Supplementary Figure 10. Lomitapide enhances the efficacy of chemotherapy in PDAC.

A and B, Representative confocal micrographs (A) and quantitation (B) showing the effects of mFOLFIRINOX (8 µM) and lomitapide (2 µM) treatment on LD levels in MiaPaCa-2 and PANC-1 cells.

C, Zero Interaction Potency (ZIP) score analysis of the synergistic effects of mFOLFIRINOX and Lomitapide treatments in control and CLSA-I PDO-1 and PDO-2.

D, The effect of lomitapide (0.5 µM) treatment on the sensitivity of control and CLSA-25 PDAC cells to Abrexane / Gemcitabine regimen. Abrexane and Gemcitabine were added at 1:20 molar ratios and the shown concentrations are Gemcitabine concentrations.

E, The effects of DGAT1 and 2 knockdowns on mFOLFIRINOX sensitivities in naïve MiaPaCa-2 and PANC-1 cells.

F and G, The effects of ATGL inhibitor (Atglstatin) treatment alone (F) or in combination with Gemcitabine (G) on cell viability in control or CLSA-25 MiaPaCa-2 and PANC-1 cells. IC50 for (F) were not determined.

H, Tumors harvested from PDX-1 and PDX-2 mice treated with vehicle control, lomitapide (10 mg/kg) mFOLFIRINOX or the combination of both.

I, representative images of ^{18}F-DG PET-CT scan from PDX-2 mice treated with vehicle control, lomitapide, mFOLFIRINOX or the combination of both.

J, bodyweight of KPC mice receiving vehicle control, lomitapide, mFOLFIRINOX or the combination of both.

K and L, Representative confocal micrograph (K) or quantitation (L) of LD staining of cryosections of tumor tissues harvested from PDX-1 and PDX-2 tumors treated with vehicle control, lomitapide (10 mg/kg) mFOLFIRINOX or the combination of both.

Data in (B) and (L) were analyzed using two sample, two-tailed t-test. Data in (J) were analyzed using two-way ANOVA followed by Tukey's multiple comparison test. **** represents 0.0001. All error bars are mean ±SD

Table S1. Correlations between clinicopathological features and G6PD expression in pancreatic cancer

	G6PD			
	**Low**	**High**	***p*-values**	**Spearman rank *r* value**
**Gender**				
Male	32	25	0.397	0.032
Female	18	25		
**Age**				
> 60	27	28	0.755	0.086
≤60	23	22		
**Tumor size (cm)[b]**				
≤3	27	19	0.041	0.205
>3	23	31		
**LN metastasis**				
N 0	41	35	<0.001	0.480
N 1	9	15		
**pTNM stage**				
I	37	24	**<0.001**	0.376
II, III	13	26		

Data are analyzed using Spearman's rank correlation test.

Table S2. Small molecule screening

**Category**	**Parameter**	**Description**
Assay	Type of assay	Cell Viability / Cytotoxicity Assay (based on Cell Counting Kit-8, CCK8)
	Target	Cell viability of naïve PDAC cells and CLSA PDAC cells
	Primary measurement	Cell viability

	Key reagents	Cell Counting Kit-8 test compounds DMSO (vehicle control)
	Assay protocol	1. Seed target cells (naïve PDAC cells, CLSA PDAC cells) onto 96-well plates at an optimal density in their respective medium; 2. Incubate the plates at 37 °C in a 5% CO_2 environment for 16 hours to allow cell attachment and adaptation; 3. Add compounds from the L5200 Anti-Metabolism disease Compound Library to the wells (with DMSO as the vehicle control) to reach a final concentration of 5 μM; 4. Incubate the cells with compounds for 48 hours; 5. Measure cell viability using the Cell Counting Kit-8 according to the assay principle
	Additional comments	The assay was designed to investigate whether CLSA could expose exploitable metabolic vulnerabilities in PDAC cells
Library	Library size	825 Compound
	Library composition	Anti-Metabolism disease Compound, focusing on metabolic targets including glycolysis/glucose uptake, nucleotide synthesis, and lipid metabolism
	Source	L5200-Anti-Metabolism disease Compound Library (TOPSCIENCE)
	Additional comments	The library covers multiple metabolic pathways, providing a basis for screening compounds that target metabolic vulnerabilities associated with CLSA
Screen	Format	96-well plate format

	Concentration(s) tested	Final concentration of 10 μM for all test compounds
	Plate controls	Vehicle control (DMSO) to account for non-specific effects of the solvent on cell viability
	Reagent/ compound dispensing system	Explorer G3 Automated Drug Screening System
	Detection instrument and software	EnVision 2105 Multimode Detection Instrument
	Assay validation/QC	Consistent incubation conditions (37 °C, 5% CO_2) and standardized CCK-8 assay procedures ensured reproducibility; screening focused on metabolic vulnerabilities to align with the library's anti-metabolism focus
	Correction factors	Not specified
	Normalization	Normalized to the vehicle control (DMSO) to eliminate the impact of solvent on cell viability and standardize the comparison of compound effects
	Additional comments	Automated compound dispensing via the Explorer G3 system guaranteed accurate and uniform delivery of test compounds
Post-HTS analysis	Hit criteria	Compounds that induced more than 70% inhibition of cell viability in MiaPaCa-2-CLSA cells
	Hit rate	~9.2% (76 positive hits out of 825 total compounds in the library)
	Additional assay(s)	1. Further functional validation of lipid metabolism-targeting hits (e.g., Lomitapide, Mevastatin, Troglitazone, Pioglitazone, Denifanstat, A939572) in naïve and CLSA PDAC cells; 2. IC50

	determination of Lomitapide (a top hit) in CLSA PDAC cells and PDOs (IC50 range: high nanomolar to low micromolar); 3. Rescue assays using zVAD-fmk (pan-caspase inhibitor), necrostatin-1 (RIPK1 inhibitor), and ferrostatin-1 (ferroptosis inhibitor) to identify the mechanism of Lomitapide-induced cell death; 4. Detection of lipid peroxidation in Lomitapide-treated control and CLSA PDAC cells
Confirmation of hit purity and structure	Not specified
Additional comments	1. Positive hits included glycolysis/glucose uptake inhibitors (e.g., PFK158, BAY-876), nucleotide synthesis inhibitors (e.g., Leflunomide, Pemetrexed acid), and lipid metabolism-targeting compounds (~30% of positive hits); 2. Lomitapide (a lipid metabolism-targeting hit) showed excellent efficacy in CLSA PDAC cells/PDOs, and its induced cell death involved apoptosis and necroptosis (not ferroptosis, confirmed by rescue assays and lipid peroxidation detection)

Table S3: Antibodies and their dilutions

Name	Company	Cat. No.	Dilution (Western blotting)	Dilution (IHC or IF)
NDUFS1	Proteintech	12444-1-AP	2500	-
SDHB	Santa Cruz	sc-271548	500	-
Rieske	Santa Cruz	sc-271609	500	-

COX2	Santa Cruz	sc-514489	500	-
G6PD	Santa Cruz	sc-373886	500	200
PGLS	Santa Cruz	sc-398833	250	200
PGD	Santa Cruz	sc-398977	1000	200
GCLC	Santa Cruz	sc-390811	500	-
GCLM	Santa Cruz	sc-55586	500	-
α-tubulin	Santa Cruz	sc-5286	20000	-
MTTP	Santa Cruz	sc-515742	500	-
GAPDH	Sigma	G8795	20000	-
Histone	Millipore	07-690	25000	-
ATF-4	cell Signaling	11815S	1000	200
Phospho-eIF2α (Ser51)	Cell signaling	9721S	1000	-
eIF2α Antibody	Cell signaling	9722S	1000	-
CK19	abcam	ab76539	500	1000
α-SMA	abcam	ab7818	500	500
FSP1	santa cruz	sc-377120	1000	-
DHODH	Proteintech	14877-1-AP	2000	-
COQ2	Thermo Fisher	PA5107103	500	-
COQ6	Santa Cruz	sc-393932	500	-
COQ9	Santa Cruz	sc-365073	1000	-
GPX4	abcam	ab252833	1000	-
PLIN2	abcam	ab52356	-	100
SLC7A11	abcam	ab307601	1000	200
FSP1	santa cruz	sc-377120	1000	
DHODH	Proteintech	14877-1-AP	2000	
COQ2	Thermo Fisher	PA5107103	500	
COQ6	Santa Cruz	sc-393932	500	
COQ9	Santa Cruz	sc-365073	1000	
GPX4	abcam	ab252833	1000	
PLIN2	abcam	ab52356		100
SLC7A11	abcam	ab307601	1000	
NFS1	Santa Cruz	sc-365308	500	
IscU1/2	Santa Cruz	sc-373694	500	
IRP2	Cell signaling	37135S	1000	

IRP1	Cell signaling	20272S	1000
TFRC	Cell signaling	13113S	5000
FTH1	Santa Cruz	sc-376594	2500
CSE	Cell signaling	30068S	1000
CBS	Cell signaling	14782S	1000

Table S4: Key regagents

Name	Company	Cat. No.
DMSO	sigma	D2650
Fetal Bovine Serum	Atlanta Biological	S11150
Penicillin/Streptomycin	Gibco	15140163
Trypsin-EDTA	Gibco	25200-056
Gibco™ DMEM, high glucose, no glutamine, no methionine, no cystine	Gibco	21-013-024
DMEM without AMINO ACID and D-Glucose	life technology	D98002710
DMEM	Cytiva	SH30243.01
LipidSpot™ 488	Biotium	#70065
SODIUM PALMITATE,	SIGMA ALDRICH INC	P9767
Bovine Serum Albumin (FFA-free)	GoldBio	57775
RIPA Lysis and Extraction Buffer	Thermo Fisher	88901
IODINE, ROUND PARTICLES, DIAMETER 1-2.5	SIGMA ALDRICH INC	57655-100G-F
1-MONOOLEOYL-RAC-GLYCEROL (C18:1,-CIS-9)	SIGMA ALDRICH INC	M7765
OLEIC ACID BIOREAGENT, SUITABLE FOR CEL	SIGMA ALDRICH INC	O1383
15 0 18 1 15 0 TG	FISHER SCIENTIFIC	NC2291691
16:0-18:1 DG 10MG	VWR INTL LLC	100125-990
3-azido-7-hydroxy Coumarin	Cayman	10596
N,N-DIISOPROPYLETHYLAMINE, BIOTECH GRAD	SIGMA ALDRICH INC	496219
Oleic Acid Alkyne	Cayman	9002078
Palmitic Acid Alkyne	Cayman	13266
Collagenase from Clostridium histolytic	SIGMA ALDRICH INC	C1639
HEXANE	SIGMA ALDRICH INC	34859
TETRAKIS(ACETONITRILE)COPPER(I) TETRAF	SIGMA ALDRICH INC	677892
16-NBD-16:0 Coenzyme A	Avanti Polar Lipids	810705P
1,2-DIOLEOYL-SN-GLYCEROL, >=97%,	SIGMA ALDRICH INC	D0138
L-GLUTAMINE-13C5,15N2 98+%13C-98+%15N	SIGMA ALDRICH INC	607983
D-Glucose (U-$^{13}C_6$, 99%)	Cambridge Isotope Laboratories	CLM-1396-10
L-Cysteine ($^{13}C_3$, 99%; ^{15}N, 99%)	Cambridge Isotope Laboratories	CNLM-3871-H-0.1
ETHYL ETHER ANH R ACS 4L	FISHER SCIENTIFIC	E1384
L-Azidohomoalanine (AHA)	Click Chemistry Tools	1066-25

Biotin-PEG4-Alkyne	Click Chemistry Tools	TA105-5
AZDye 488 Alkyne	Click Chemistry Tools	1277-1
TBTA	Click Chemistry Tools	1061-100
Cycloheximide (CHX)	SIGMA ALDRICH INC	C7698
DAPI (4',6-Diamidino-2-Phenylindole, Dihydrochloride)	Thermo Fisher	D1306
Formalin 10%	FISHER SCIENTIFIC	SF100-4
Crystal violet solution	Eng Scientific	6100
Propidium iodide	SIGMA ALDRICH INC	P4170
PVDF Transfer Membranes	Millipore	IPVH00010
Methanol	FISHER SCIENTIFIC	BP11054
Phosphatase inhibitor cocktail	Thermo Fisher	88667
Protease Inhibitor Cocktail	SIGMA ALDRICH INC	P8340
Dispase II	SIGMA ALDRICH INC	D4693
L-Glutamine	Corning	61-030-RM
L-Methionine	ACROS	AC166160250
Sodium Pyruvate	SIGMA ALDRICH INC	P5280
L-Cytine 2HCl	SIGMA ALDRICH INC	C7777
D-Luciferin Sodium Salt	Regis Tech	103404-75-7
Sucrose	Millipore	573113
DNase I, RNase-free	fisher	EN0525
RiboLock Rnase Inhibitor	fisher	EO0382
Matrigel	Corning	CLS354248
TLC plates, Silica gel 60 with concentrating zone 2.5 x 20 cm	Merk	1118450001
pENTR/D-TOPO Cloning Kit	Thermo Fisher	K240020
Gateway™ LR Clonase™ II Enzyme mix	Thermo Fisher	11791020
High-Capacity cDNA Reverse Transcription Kit	Thermo Fisher	4368814
SYBR™ Green PCR Master Mix	Thermo Fisher	4367659
Aconitase Activity Assay	Sigma	MAK051
CellTiter-Glo® Luminescent Cell Viability Assay	Promega	G7572
Seahorse XF Palmitate Oxidation Stress Test Kit	Agilent Technologies Inc	103693-100
Seahorse XF Cell Mito Stress Test Kit	Agilent Technologies Inc	103015-100
Seahorse XF Glycolysis Stress Test Kit	Agilent Technologies Inc	103020-100
Oasis WAX 1 cc Vac Cartridge, 30 mg Sorbent per Cartridge, 30 µm, 100/pk	Waters	186002489
Lipofectamine™ RNAiMAX Transfection Reagent	Thermo Fisher Scientific	13778030
Lactate Assay Kit	Sigma	MAK064
RNeasy Kit	QIAGEN	74106
FITC-dextran	MCE	HY-128868
EIPA	MCE	HY-101840
L-Serine (1-13C,99%)	Cambridge Isotope	CLM-1573-0.25

Table S5: siRNA and shRNA sequence

hs.Ri.DGAT1.13.1-SEQ1	rGrGrUrCrUrUrArCrUrGrGrUrUrGrArGrUrCrUrArUrCrACT
hs.Ri.DGAT1.13.1-SEQ2	rArGrUrGrArUrArGrArCrUrCrArArCrCrArGrUrArArGrArCrCrArC
hs.Ri.DGAT2.13.1-SEQ1	rArGrArUrUrCrUrGrGrArUrGrUrGrArGrGrArArGrArGrATC
hs.Ri.DGAT2.13.1-SEQ2	rGrArUrCrUrCrUrUrCrCrUrCrArCrArUrCrCrArGrArArUrCrUrArG
hs.Ri.G6PD.13.1-SEQ1	rGrGrUrCrArArGrGrUrGrUrUrGrArArArUrGrCrArUrCrUCA
hs.Ri.G6PD.13.1-SEQ2	rUrGrArGrArUrGrCrArUrUrUrCrArArCrArCrCrUrUrGrArCrCrUrU
hs.Ri.G6PD.13.2-SEQ1	rCrUrGrArCrCrUrArCrGrGrCrArArCrArGrArUrArCrArAGA
hs.Ri.G6PD.13.2-SEQ2	rUrCrUrUrGrUrArUrCrUrGrUrUrGrCrCrGrUrArGrGrUrCrArGrGrU
SLC7A11 shRNA targeting sequence	CCTGTCACTATTTGGAGCTTT
G6PD shRNA targeting sequence	GTCGTCCTCTATGTGGAGAAT
COQ2-1 shRNA targeting sequence	CTTGGGAATTTGTGGAAAGAA
COQ2-2 shRNA targeting sequence	TGGGACCAGGACTATGATAAA

Table S6: qPCR primer sequence

hDGAT1-F	CTGGGAGCTGAGGTGC
hDGAT1-R	CACACACCAGTTCAGGATGC
hDGAT2-F	TCTGGGAGATGGGGCACTG
hDGAT2-R	GCTGCTTTTCCACCTTGGAC
hG6PD-F	CACCATGGCAGAGCAGGTGGCC
hG6PD-R	TCAGAGCTTGTGGGGGTTCACCCA
hPGLS-F	CCGGTTTTCGACCTGCTGAT
hPGLS-R	GGAGCCACAATCTTCTCCCG
hPGD-F	AACTCTTGGCCAAACCAGGG
hPGD-R	GGAGCCCATCAGGCATTGTAT

h.b-actin-F	CTTCGCGGGCGACGAT
h.b-actin-R	TAGGAATCCTTCTGACCCATGC
hGAPDH-F	GTCTCCTCTGACTTCAACAGCG
hGAPDH-R	ACCACCCTGTTGCTGTAGCCAA
