# Supplementary material for: Adaptation to cystine limitation stress confers a targetable lipid metabolism vulnerability in pancreatic ductal adenocarcinoma
Source: Nat Commun. 2025 Dec 31;17:1343. doi: 10.1038/s41467-025-68099-0 (PMC12872613; doi:10.1038/s41467-025-68099-0)
Supplement: Supplementary file 2 — Reporting Summary [file 41467_2025_68099_MOESM2_ESM.pdf]

nature portfolio

Corresponding author(s): Shengyu Yang, Jihui Hao, Xiuchao Wang

Last updated by author(s): 09/28/2025

Reporting Summary

Nature Portfolio wishes to improve the reproducibility of the work that we publish. This form provides structure for consistency and transparency in reporting. For further information on Nature Portfolio policies, see our Editorial Policies and the Editorial Policy Checklist.

Statistics

For all statistical analyses, confirm that the following items are present in the figure legend, table legend, main text, or Methods section.

n/a	Confirmed	
☐	☒	The exact sample size (n) for each experimental group/condition, given as a discrete number and unit of measurement
☐	☒	A statement on whether measurements were taken from distinct samples or whether the same sample was measured repeatedly
☐	☒	The statistical test(s) used AND whether they are one- or two-sided<br>*Only common tests should be described solely by name; describe more complex techniques in the Methods section.*
☒	☐	A description of all covariates tested
☐	☒	A description of any assumptions or corrections, such as tests of normality and adjustment for multiple comparisons
☐	☒	A full description of the statistical parameters including central tendency (e.g. means) or other basic estimates (e.g. regression coefficient) AND variation (e.g. standard deviation) or associated estimates of uncertainty (e.g. confidence intervals)
☐	☒	For null hypothesis testing, the test statistic (e.g. F, t, r) with confidence intervals, effect sizes, degrees of freedom and P value noted<br>*Give P values as exact values whenever suitable.*
☒	☐	For Bayesian analysis, information on the choice of priors and Markov chain Monte Carlo settings
☒	☐	For hierarchical and complex designs, identification of the appropriate level for tests and full reporting of outcomes
☐	☒	Estimates of effect sizes (e.g. Cohen's d, Pearson's r), indicating how they were calculated

Our web collection on statistics for biologists contains articles on many of the points above.

Software and code

Policy information about availability of computer code

Data collection	Flow cytometry data were acquired using BD Canto10. Fluorescent images were captured in a confocal microscope Leica SP8. Optical density intensities were measured in FlexStation 3 (Molecular Devies). OCR and ECAR data were acquired in XFe24 Extracellular Flux Analyzer (Agilent Seahorse Bioscience).
Data analysis	GraphPad Prism 9 was used for bar graphs, Heat Map,Viability curve output and statistical analysis; FlowJo V10 was used for flow cytometry data analysis. The LC-MS/MS data were analyzed using Sciex OS 3.0 software.

For manuscripts utilizing custom algorithms or software that are central to the research but not yet described in published literature, software must be made available to editors and reviewers. We strongly encourage code deposition in a community repository (e.g. GitHub). See the Nature Portfolio guidelines for submitting code & software for further information.

Data

Policy information about availability of data

All manuscripts must include a data availability statement. This statement should provide the following information, where applicable:

- Accession codes, unique identifiers, or web links for publicly available datasets
- A description of any restrictions on data availability
- For clinical datasets or third party data, please ensure that the statement adheres to our policy

Data availability: Metabolomics and lipidomics data are being deposited to National Metabolomics Data Repository (NMDR) (accession: PR002249; http://dx.doi.org/10.21228/M8XC02) and are publicly available as of the date of publication.

Original western blot images are being included as source data associated with this manuscript and are publicly available as of the date of publication.
Code availability: There was no unique code associated with this paper.

Research involving human participants, their data, or biological material

Policy information about studies with human participants or human data. See also policy information about sex, gender (identity/presentation), and sexual orientation and race, ethnicity and racism.

Reporting on sex and gender	De-identified human PDAC specimens were used in the studies. Table S1 reported the the de-aggregated data.
Reporting on race, ethnicity, or other socially relevant groupings	NA
Population characteristics	The age and gender characteristics of the PDAC patients are reported in Table S1.
Recruitment	The tissue specimens were from PDAC patients treated at the Tianjin Medical University Cancer Institute and Hospital, China. These patients signed a written informed consent for the use of their specimens and disease related information in future reserach.
Ethics oversight	In accordance with the requirements of the Ethics Committee of Tianjin Medical University Cancer Institute and Hospital, China, and the recognized ethical guidelines of the Declaration of Helsinki, all patients provided written informed consent for the use of their specimens and disease-related information in future research (Approval No.: EK20220153).

Note that full information on the approval of the study protocol must also be provided in the manuscript.

Field-specific reporting

Please select the one below that is the best fit for your research. If you are not sure, read the appropriate sections before making your selection.

☒ Life sciences ☐ Behavioural & social sciences ☐ Ecological, evolutionary & environmental sciences

For a reference copy of the document with all sections, see nature.com/documents/nr-reporting-summary-flat.pdf

Life sciences study design

All studies must disclose on these points even when the disclosure is negative.

Sample size	No sample size calculations were performed. The sample size of the experiments was chosen based on previous experience in the lab.
Data exclusions	No samples or animals were excluded from the data analyses.
Replication	Multiple independent repeats were included for related experiments. Each experiment was performed for at least twice to make sure similar results are reproducible.
Randomization	6-7-week-old female NSG, C57BL/6J, BALB/c Nude mice were chosen as xenograft hosts and randomly allocated into experimental groups. KPC mice (C57BL/6Smoc-Trp53 em4(R172H)Kras em4(LSL-G12D)Tg(Pdx1-cre)Smoc mice, 8 weeks old, male) were randomized into 4 groups for drug treatment.
Blinding	For cell-based experiments, western blotting, immunostaining and FACS, data collection was conducted blindly. Measurement for cell viability, FACS, photo capture and histological analysis were performed by different individuals who were blinded to the experimental groups. Mass spectrometry analysis was blinded prior to analysis.

Reporting for specific materials, systems and methods

We require information from authors about some types of materials, experimental systems and methods used in many studies. Here, indicate whether each material, system or method listed is relevant to your study. If you are not sure if a list item applies to your research, read the appropriate section before selecting a response.

Materials & experimental systems

n/a	Involved in the study	
	x	Antibodies
	x	Eukaryotic cell lines
x		Palaeontology and archaeology
	x	Animals and other organisms
	x	Clinical data
x		Dual use research of concern
x		Plants

Methods

n/a	Involved in the study	
x		ChIP-seq
	x	Flow cytometry
x		MRI-based neuroimaging

Antibodies

Antibodies used

NDUFS1 Proteintech 12444-1-AP 1:2500
SDHB Santa Cruz sc-271548 1:500
Rieske Santa Cruz sc-271609 1:500
COX2 Santa Cruz sc-514489 1:500
G6PD Santa Cruz sc-373886 1:500
PGLS Santa Cruz sc-398833 1:250
PGD Santa Cruz sc-398977 1:1000
CTNS Proteintech 13085-1-AP 1:2500
GCLC Santa Cruz sc-390811 1:500
GCLM Santa Cruz sc-55586 1:500
α-tubulin Santa Cruz sc-5286 1:20000
MTP Santa Cruz sc-515742 1:500
GAPDH Sigma G8795 1:20000
Histone Millipore 07-690 1:25000
ATF-4 cell Signaling 11815S 1:1000
Phospho-eIF2α (Ser51) Cell signaling 9721S 1:1000
eIF2α Cell signaling 9722S 1:1000
CK19 Abcam ab76539 1:500
α-SMA Proteintech 14395-1-AP 1:500
FSP1 Santa cruz sc-377120 1:1000
DHODH Proteintech 14877-1-AP 1:2000
COQ2 Thermo Fisher PA5107103 1:500
COQ6 Santa Cruz sc-393932 1:500
COQ9 Santa Cruz sc-365073 1:1000
GPX4 Abcam ab252833 1:1000
PLIN2 Abcam ab52356 1:100
SLC7A11 Abcam ab307601 1:1000
NFS1 Santa Cruz sc-365308 1:500
IscU1/2 Santa Cruz sc-373694 1:500
IRP2 Cell signaling 37135S 1:1000
IRP1 Cell signaling 20272S 1:1000
TFRC Cell signaling 13113S 1:5000
FTH1 Santa Cruz sc-376594 1:2500
CSE Cell signaling 30068S 1:1000
CBS Cell signaling 14782S 1:1000

Validation

All antibodies used in our study have been validated and detailed information could be found on the website from manufactures as listed below.
https://www.ptglab.com/products/NDUFS1-Antibody-12444-1-AP
https://www.scbt.com/p/sdhb-antibody-g-10
https://www.scbt.com/p/rieske-fes-antibody-a-5
https://www.scbt.com/p/cox2-antibody-d-5
https://www.scbt.com/p/g6pd-antibody-g-12
https://www.scbt.com/p/pgls-antibody-g-7
https://www.scbt.com/p/pgd-antibody-g-2
https://www.ptglab.com/products/CTNS-Antibody-13085-1-AP.htm
https://www.scbt.com/p/gamma-gcsc-antibody-h-5
https://www.scbt.com/p/gamma-gcsm-antibody-e-4
https://www.scbt.com/p/alpha-tubulin-antibody-b-7
https://www.scbt.com/p/mtp-antibody-c-1

https://www.sigmaaldrich.com/US/en/product/sigma/g8795
https://www.sigmaaldrich.com/US/en/product/mm/07690
https://www.cellsignal.com/products/primary-antibodies/atf-4-d4b8-rabbit-mab/11815
https://www.cellsignal.com/products/primary-antibodies/phospho-eif2a-ser51-antibody/9721
https://www.cellsignal.com/products/primary-antibodies/eif2a-antibody/9722
https://www.abcam.cn/products/primary-antibodies/cytokeratin-19-antibody-epr1579y-ab76539
https://www.ptgcn.com/products/ACTA2-Antibody-14395-1-AP.htm
https://www.scbt.com/zh/p/amid-antibody-b-6
https://www.ptgcn.com/products/DHODH-Antibody-14877-1-AP.htm
https://www.thermofisher.cn/cn/zh/antibody/product/COQ2-Antibody-Polyclonal/PA5-107103
https://www.scbt.com/zh/p/coq6-antibody-h-1
https://www.scbt.com/zh/p/coq9-antibody-g-4
https://www.abcam.cn/products/primary-antibodies/glutathione-peroxidase-4-antibody-1b4-ab252833
https://www.abcam.cn/products/primary-antibodies/perilipin-2-antibody-ab52356
https://www.abcam.cn/products/primary-antibodies/xct-antibody-epr27115-64-ab307601
https://www.scbt.com/zh/p/nfs1-antibody-b-7
https://www.scbt.com/zh/p/iscu1-2-antibody-d-6
https://www.cellsignal.cn/products/primary-antibodies/irp2-d6e6w-rabbit-mab/37135
https://www.cellsignal.cn/products/primary-antibodies/irp1-d6s4j-rabbit-mab/20272
https://www.cellsignal.cn/products/primary-antibodies/transferrin-receptor-cd71-d7g9x-xp-rabbit-mab/13113
https://www.scbt.com/zh/p/ferritin-heavy-chain-antibody-b-12
https://www.cellsignal.cn/products/primary-antibodies/cystathionine-g-lyase-d4e9j-rabbit-mab/30068
https://www.cellsignal.cn/products/primary-antibodies/cbs-d8f2p-rabbit-mab/14782

Eukaryotic cell lines

Policy information about cell lines and Sex and Gender in Research

Cell line source(s)	The MiaPaCa-2(CRL-1420), PANC-1 (CRL-1469), SW-1990 (CRL-2172), Capan-2 (HTB-80) cell lines were obtained from the ATCC. Pan02 cell was a gift from Dr. Shari Pilon-Thomass (Moffitt Cancer Center). KPC cell line was derived from KPC PDAC model as described in the manuscript.
Authentication	MiaPaCa-2, PANC-1, SW-1990, Capan-2 were authenticated through STR analysis.
Mycoplasma contamination	All cell lines tested negative for mycoplasma contamination.
Commonly misidentified lines (See ICLAC register)	No ICLAC cell line was used in this study.

Animals and other research organisms

Policy information about studies involving animals; ARRIVE guidelines recommended for reporting animal research, and Sex and Gender in Research

Laboratory animals	6-7-week-old NSG, C57BL/6J, BALB/c nude mice were purchased from the Jackson Lab. Mice were housed under specific-pathogen-free conditions with a 12 h light-12 h dark cycle. KPC mice (C57BL/6Smoc-Trp53 em4(R172H)Kras em4(LSL-G12D)Tg(Pdx1-cre)Smoc mice, 8 weeks old, male) were purchased from Shanghai Model Organisms Center. The ambient temperature was 21-23°C, with 45% humidity and the mice had ad libitum access to water and food.
Wild animals	No wild animals involved in this study.
Reporting on sex	Female mice were used for orthotopic xenograft and experiments due to their ease of handling in this study. Male KPC mice was used because female mice were not available from Shanghai Model Organisms Center. Sex was not a factor considered in the study design.
Field-collected samples	No sample collected from field was used in this study.
Ethics oversight	All the xenograft model experiments were conducted in compliance with a protocol approved by the IACUC committee at the Penn State College of Medicine or Tianjin Cancer Hospital. The study adheres to all relevant ethical regulations pertaining to animal research.

Note that full information on the approval of the study protocol must also be provided in the manuscript.

Clinical data

Policy information about clinical studies
All manuscripts should comply with the ICMJE guidelines for publication of clinical research and a completed CONSORT checklist must be included with all submissions.

Clinical trial registration	NA

Study protocol	In accordance with the requirements of the Ethics Committee of Tianjin Medical University Cancer Institute and Hospital, China, and the recognized ethical guidelines of the Declaration of Helsinki, all patients provided written informed consent for the use of their specimens and disease-related information in future research (Approval No.: EK20220153).
Data collection	NA
Outcomes	NA

Plants

Seed stocks	NA
Novel plant genotypes	NA
Authentication	NA

Flow Cytometry

Plots

Confirm that:

- [x] The axis labels state the marker and fluorochrome used (e.g. CD4-FITC).
- [x] The axis scales are clearly visible. Include numbers along axes only for bottom left plot of group (a 'group' is an analysis of identical markers).
- [x] All plots are contour plots with outliers or pseudocolor plots.
- [x] A numerical value for number of cells or percentage (with statistics) is provided.

Methodology

Sample preparation	For cell death analysis, collected cells were stained with 5 µg/ml propidium iodide (PI, Sigma, P4170) and the percentage of PI-positive dead cell population was analyzed by the flow cytometer, Canto10 flow cytometer (BD). Then, cells were collected and subjected to flow cytometry analysis by he flow cytometer, Canto10 flow cytometer (BD). For glucose up-take assay, The cells were incubated with 50 µM 2-NBDG and incubated at 37 °C for 2 h.
Instrument	Canto10 flow cytometer (BD)
Software	FlowJo_V10 software was used for data analysis
Cell population abundance	At least 10,000 cells were analyzed for data collection.
Gating strategy	Initial cell population gating (SSC-Area VS SSC-Height) was adopted to make sure doublet exclusion and only single cell was used for analysis. An identical cell gating strategy was applied to all samples analyzed at the same time.

- [x] Tick this box to confirm that a figure exemplifying the gating strategy is provided in the Supplementary Information.
